# Supplementary material for: A fault-intrusion-tolerant system and deadline-aware algorithm for scheduling scientific workflow in the cloud
Source: PeerJ Comput Sci. 2021 Nov 2;7:e747. doi: 10.7717/peerj-cs.747 (PMC8576554; doi:10.7717/peerj-cs.747)
Supplement: Supplemental Information 6 [file peerj-cs-07-747-s006.zip › Myproject1.1/docs/org/cloudbus/cloudsim/network/class-use/TopologicalGraph.html]

Uses of Class org.cloudbus.cloudsim.network.TopologicalGraph


JavaScript is disabled on your browser.


- Overview
- Package
- Class
- Use
- Tree
- Deprecated
- Index
- Help

- Prev
- Next

- Frames
- No Frames

- All Classes

## Uses of Class org.cloudbus.cloudsim.network.TopologicalGraph

- Packages that use TopologicalGraph

  | Package | Description |
  | --- | --- |
  |  |  |
  | --- | --- |
  | org.cloudbus.cloudsim |  |
  | org.cloudbus.cloudsim.network |  |
- - ### Uses of TopologicalGraph in org.cloudbus.cloudsim

    Fields in org.cloudbus.cloudsim declared as TopologicalGraph

    | Modifier and Type | Field and Description |
    | --- | --- |
    |  |  |
    | --- | --- |
    | `protected static TopologicalGraph` | NetworkTopology.`graph` |
  - ### Uses of TopologicalGraph in org.cloudbus.cloudsim.network

    Methods in org.cloudbus.cloudsim.network that return TopologicalGraph

    | Modifier and Type | Method and Description |
    | --- | --- |
    |  |  |
    | --- | --- |
    | `TopologicalGraph` | GraphReaderBrite.`readGraphFile(java.lang.String filename)` this method just reads the file and creates an TopologicalGraph object |
    | `TopologicalGraph` | GraphReaderIF.`readGraphFile(java.lang.String filename)` this method just reads the file and creates an TopologicalGraph object |

    Constructors in org.cloudbus.cloudsim.network with parameters of type TopologicalGraph

    Constructor and Description

    `DelayMatrix_Float(TopologicalGraph graph,
    boolean directed)`

    this constructor creates an correct initialized Float-Delay-Matrix

- Overview
- Package
- Class
- Use
- Tree
- Deprecated
- Index
- Help

- Prev
- Next

- Frames
- No Frames

- All Classes
